# Oral Argument: Sublingual Findings Challenge Key Assumptions about BPA Exposure

**DOI:** 10.1289/ehp.121-a257

**Published:** 2013-08-01

**Authors:** Julia R. Barrett

**Affiliations:** Julia R. Barrett, MS, ELS, a Madison, WI–based science writer and editor, has written for *EHP* since 1996. She is a member of the National Association of Science Writers and the Board of Editors in the Life Sciences.

Key assumptions about bisphenol A (BPA) exposure and bioavailability may be off base, according to a new report in *EHP* that questions the traditional interpretation of biomonitoring data underlying current risk assessments of the chemical.^1^ Laboratory research suggests that BPA, a widely used chemical for polycarbonate plastics and other products, is an endocrine disruptor with potential adverse health effects involving reproduction, metabolism, and cancer.^2^^,^^3^

Median daily intake of the chemical through the diet is estimated to be 0.01–0.12 µg/kg body weight, based on urinary concentrations of BPA metabolites.^4^ These metabolites, particularly BPA glucuronide (BPAG), are not biologically active like the parent compound. Nearly all ingested BPA has been thought to be absorbed in the small intestine and rapidly converted to BPAG in the liver prior to bodywide distribution.^5^^,^^6^

Given the estimated daily intake and assumption of rapid conversion, the European Food Safety Authority set a tolerable daily intake of BPA at 0.05 mg/kg.^1^ However, the current study indicates that BPA can be completely absorbed directly into the bloodstream from the mouth, thus bypassing early rapid metabolism and remaining biologically active for an extended period of time.

“Most previous studies have relied on the gavage method, where BPA is distributed directly into the gut,” says Laura Vandenberg, a postdoctoral fellow at the Tufts University Center for Regenerative and Developmental Biology, who was not involved in the study. “This isn’t how food actually enters our bodies. We chew it, move it around in our mouths, and it interacts with numerous surfaces—our tongue, cheeks, etc.—before it enters the stomach.” Gavage studies suggest there should not be detectable levels of unmetabolized BPA in the blood after exposure, yet dozens of other studies report otherwise, she says.

The researchers compared the bioavailability of BPA given sublingually (under the tongue) versus intravenously or by gavage. In one experiment 6 dogs received a dose of 5 mg/kg first intravenously, then a week later sublingually either all at once or one drop at a time. In another experiment, conducted in three parts, the same dogs received a dose of 0.05 mg/kg first intravenously, then sublingually, and finally a 20-mg/kg dose delivered by gavage. After each administration, blood samples were collected to measure BPA and BPAG and calculate various parameters such as maximum concentration, time to maximum concentration, and mean residence time.^1^

BPA was readily absorbed from both the mouth and the gut, but bioavailability throughout the body differed significantly depending on the absorption site. Over the 2 hours following dosing, 5 mg/g and 0.05 mg/kg sublingual BPA yielded ratios of biologically inactive BPAG to biologically active BPA of 1:1–13:1 and 1:1–6:1, respectively, compared with 237:1–634:1 for the dose placed directly in the stomach.^1^

**Figure:**
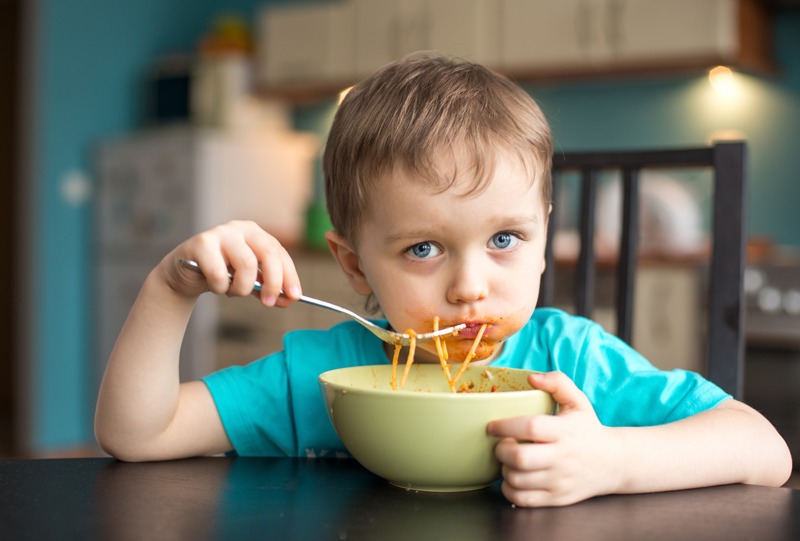
If confirmed, new findings on sublingual BPA dosing could have important implications for human exposures to BPA. © Shutterstock.com

“The sublingual route is a rather well-known route of absorption for those who are involved in the development of drugs but not for those working with contaminants in general and BPA in particular,” says study coauthor Pierre-Louis Toutain, a professor of physiology and therapeutics at the National Veterinary School of Toulouse, France. He says dogs were chosen as the study animals because their oral tissue closely resembles that of humans.

The sublingual dosing used in the study may present different conditions from the exposure that occurs while eating or drinking. It also does not reflect potential nonfood exposures from sources such as dust, cigarette filters, thermal papers, and dental sealants that previous research has suggested occur.^7^ However, “the authors have shown very clearly that when BPA comes into contact with the mucosa under the tongue, it can be rapidly absorbed into the bloodstream. When this happens, it enters the bloodstream without being metabolized,” says Vandenberg. Additionally, this study may explain previous biomonitoring reports of plasma BPA levels deemed impossible or incorrect based on assumptions about gut-only absorption. This is important because plasma levels found in biomonitoring studies are similar to those found to cause adverse health effects in animal studies.^7^

Urinary BPAG serves as an index of external BPA exposure in biomonitoring studies, but it tells us nothing about the route of absorption, says Toutain. Given the researchers’ findings, he says it would be unacceptable to assume that only a negligible fraction of BPAG circulated as active BPA before it was converted to the inactive form measured in urine. He says, “It is clear that our data suggesting that BPA bioavailability can be high should raise some questions and possibly lead some agencies to reconsider their risk analysis on BPA.”
